# Phantom‐based assessment of a modified 4D‐MRI protocol for abdominal tumor motion tracking: Effects of sequence parameters and modality comparison

**DOI:** 10.1002/acm2.70446

**Published:** 2025-12-29

**Authors:** Morgan Aire, David Solis, Reagan Dugan, Olivia Magneson, Krystal Kirby

**Affiliations:** ^1^ Department of Physics and Astronomy Louisiana State University Baton Rouge Louisiana USA; ^2^ Department of Radiation Oncology Mary Bird Perkins Cancer Center Baton Rouge Louisiana USA; ^3^ Department of Radiology University of Chicago Chicago Illinois USA

**Keywords:** abdominal imaging, 4D‐CT, liver cancer, 4D‐MRI, radial VIBE, radiotherapy planning, self‐navigating

## Abstract

**Purpose:**

To investigate the effects of targeted modifications to a clinical 4D‐MR pulse sequence on displacement accuracy for abdominal tumor motion tracking, and to compare its performance with established 4D‐MR and 4D‐CT protocols using a motion phantom driven by clinically representative respiratory waveforms.

**Methods:**

A commercially available MRI‐compatible motion phantom was used to simulate abdominal tumor motion driven by both sinusoidal and patient‐derived respiratory waveforms. Imaging was performed on a 1.5T MRI scanner using a 3D radial stack‐of‐stars sequence for 4D‐MR imaging and a clinical CT scanner. Four acquisition parameters, slice thickness, acquisition orientation, number of radial views, and number of respiratory bins, were systematically varied. Displacement measurements were performed using line intensity profiles extracted from coronal slices and analyzed based on full‐width‐at‐half‐maximum calculations. Comparisons were made against both ground‐truth programmed displacements and clinical 4D‐CT measurements. Additional displacement measurement comparisons between a coronal 10‐bin 4D‐MR protocol and an axial 5‐bin 4D‐MR and 4D‐CT scans were evaluated on both sinusoidal and patient‐derived respiratory traces. Organ displacement measurements between the MR protocols were compared on compression‐belt patients as a preliminary study of the in vivo comparison.

**Results:**

The modified 4D‐MR protocol, incorporating ten respiratory bins and coronal acquisition, significantly improved displacement accuracy relative to the clinical axial MR protocol. For sinusoidal waveforms, displacement differences between coronal MR and CT were comparable (*p* > 0.05), being consistently within 0.5 mm of each other, while axial MR underestimated displacement by more than 1 mm across amplitudes and breathing periods (*p* < 0.001). Linear mixed‐effects modeling of patient‐derived respiratory waveforms showed that the coronal MR protocol outperformed the axial MR protocol by 1.70 mm for regular and 3.10 mm for irregular patterns (*p* < 0.001), with no significant differences observed between coronal MR and CT.

**Conclusions:**

A modified 4D‐MRI protocol incorporating optimized acquisition parameters demonstrated displacement accuracy equivalent to 4D‐CT in a 1D motion phantom, including under patient‐derived respiratory conditions. These results support the clinical feasibility of 4D‐MR as a radiation‐free alternative for motion‐resolved imaging in select cases, with further validation in multi‐directional motion and in vivo settings recommended.

## 1 INTRODUCTION

Radiation treatment planning aims to precisely deliver the prescribed dose to tumor volumes while minimizing exposure to adjacent healthy tissues. To achieve this objective, advanced imaging techniques are essential for accurate target and organs at risk (OARs) delineation.^1^ For abdominal lesions, particularly in the liver, pancreas, and kidneys, respiratory‐induced motion during free‐breathing image acquisition can introduce significant positional uncertainties, potentially compromising treatment accuracy.^2^, ^3^, ^4^, ^5^, ^6^, ^7^, ^8^, ^9^, ^10^, ^11^, ^12^, ^13^, ^14^, ^15^ Four‐dimensional computed tomography (4D‐CT) imaging is routinely acquired to quantify and characterize such respiratory motion to inform treatment planning.^16^, ^17^, ^18^, ^19^ Based on the magnitude and characteristics of tumor displacement assessed through 4D‐CT, motion management strategies^7^, ^17^, ^20^ are implemented in accordance with respiratory management guidelines provided by the AAPM Task Group 76 (TG‐76).^21^

While 4D‐CT provides sufficient anatomical detail for thoracic tumors due to the inherent contrast between air‐filled lung tissue and solid tumors, its utility is reduced for abdominal tumors. In the liver, for example, tumor visualization in CT is often compromised by low soft‐tissue contrast, making the tumor indistinguishable from the surrounding lung parenchyma. As a result, motion estimates are inferred from displacements of adjacent anatomical landmarks or the entire organ, which can introduce uncertainty in tumor localization and motion characterization.^3^, ^22^, ^23^, ^24^ Additionally, 4D‐CT suffers from incomplete, duplicate, and overlapping artifacts which affect tumor size and location and can be intensified by irregular breathing patterns.^25^, ^26^, ^27^, ^28^, ^29^, ^30^ Magnetic Resonance Imaging (MRI) provides the soft tissue visualization lacking in CT, offering a promising alternative for direct visualization of abdominal tumors.^31^, ^32^ Due to the long acquisition time, irregularities in breathing patterns may average out. Recently, clinical four‐dimensional MR (4D‐MR) techniques marketed for radiation therapy planning, such as a 3D radial stack‐of‐stars T1‐weighted (T1w) acquisition, have enabled amplitude‐based respiratory binning to resolve motion through the allocation of radial views into different motion‐averaged bins based on the self‐navigation respiratory signal.^32^, ^33^, ^34^ This is contrasted with the utilization of either amplitude‐ or phase‐based binning in 4D‐CT, where individual images are sorted into the correct respiratory bins. Although binning strategies differ, 4D‐MR is a potential surrogate for 4D‐CT in treatment planning workflows.

Recent studies have compared 4D‐MR and 4D‐CT in terms of accuracy in characterizing tumor displacement throughout the respiratory cycle, primarily using large‐amplitude phantom motions^35^, ^36^ of at least 30 mm. These demonstrated that MR‐derived displacement measurements are generally comparable or slightly underestimated relative to CT‐based measurements. Given that patients commonly exhibit smaller and more variable respiratory‐induced abdominal tumor motion amplitudes ranging from 5–24 mm with a majority of motion in the craniocaudal direction,^37^, ^38^ further study is needed to assess the robustness of 4D‐MR in clinically relevant scenarios.

Studies that used patient‐specific respiratory waveforms rather than idealized sinusoidal patterns similarly reported minimal differences between motion estimates between CT and MR. However, these investigations primarily assessed waveform fidelity rather than absolute displacement accuracy.^39^ Regarding sequence parameter optimization, existing literature has predominately focused on respiratory trajectory agreement, with one analyzing the effect of number of bins on displacement accuracy at set points in time. They looked at the entire trace or the trace at each point in time to assess overall agreement to 4D‐CT and ground‐truth. Without pulling the raw data, the clinical 4D‐MR sequence does not sort by exact time points due to an amplitude‐based binning algorithm. These studies generally concluded that bin number variations have minimal impact on displacement measurements over the course of the respiratory cycle.^35^ However, other sequence parameters, such as the number of radial views and acquisition orientation, have not been systematically evaluated. Both factors may substantially influence spatial resolution, artifact burden, and ultimately, the accuracy of tumor motion characterization in 4D‐MR.

Increasing the number of views improves image quality by reducing undersampling artifacts and improving signal‐to‐noise ratio (SNR) but comes at the cost of longer acquisition times. In contrast to 4D‐CT, which is restricted to axial acquisition, MR supports flexible selection of imaging planes. The radial stack‐of‐stars sequence supports acquisition in both coronal and axial orientations, with in‐plane radial blades and through‐plane stacking. In an axial acquisition, blades rotate in the transverse plane, and the sequence is more sensitive to motion along the superior‐inferior (SI) axis, aligning with the shared axis of rotation of all spokes. The orientation direction may therefore influence the degree to which respiratory‐induced displacements are captured and the extent of through‐plane artifacts such as blurring and slice misregistration. These factors are particularly relevant for abdominal imaging, where respiratory motion is a major source of image degradation.

This study investigates the influence of four key 4D‐MR acquisition parameters, slice thickness, acquisition orientation, number of radial views, and number of respiratory bins, on the accuracy of motion displacement measurements. Targeted adjustments to these parameters can enhance agreement between measured and reference displacements, thereby improving the clinical utility of 4D‐MR in motion‐resolved radiotherapy planning. The evaluation was conducted using a commercially available MR‐compatible motion phantom with both sinusoidal and patient‐derived respiratory waveforms. Smaller amplitudes, critical for motion management technique selection, as well as clinically representative breathing periods and contrast characteristics were used in this study. Following parameter evaluation, the refined 4D‐MR protocol was quantitatively compared to existing clinical MR and CT protocols to assess relative displacement accuracy. Preliminary in‐vivo displacement agreement between modalities was conducted on abdominal compression belt patients to test phantom agreement with a realistic condition. This work explores the effects of different sequence parameters on displacement accuracy and image quality across progressively realistic conditions, from controlled phantom experiments to patient‐derived respiratory patterns and initial in‐vivo imaging, supporting 4D‐MR's potential as a viable alternative to 4D‐CT in abdominal radiotherapy.

## 2 MATERIALS AND METHODS

This study protocol was reviewed and approved by the Institutional Review Board for retrospective use of imaging data and patient breathing waveforms obtained during 4D‐CT acquisition. Manuscript editing was assisted by ChatGPT 4.5 (OpenAI, San Francisco, CA, USA).

### 2.1 Phantom and experimental setup

For abdominal tumor motion simulation, the Quasar MRI4D Motion Phantom (Modus Medical Devices, Ontario, Canada) was utilized for both MR and CT imaging. The phantom incorporates a cylindrical insert containing a centrally fixed 30 mm diameter plastic sphere filled with a gadolinium‐based contrast agent (Gadovist, Bayer, Whippany, NJ, USA). The concentration of contrast material was selected to achieve a signal intensity approximately 1.5 times greater than that of the surrounding water on T1‐weighted MR imaging, simulating expected contrast between a lesion and healthy tissue. The sphere was programmed to oscillate along the superior‐inferior axis according to predefined respiratory waveforms.

### 2.2 Sinusoidal waveforms

Sinusoidal waveforms at 6 and 10 mm displacements were repeated throughout parameter comparison acquisitions, corresponding to the minimum motion thresholds used at the clinic for internal target volume (ITV) expansion and breath‐hold treatment protocols, respectively. A nominal breathing period of 4 s was used to approximate average clinical respiratory periods. Additional acquisitions were performed at 2.4 s and 12 s breathing periods to evaluate displacement accuracy across a spectrum of clinically observed fast, normal, and slow respiratory patterns. For comparison after the creation of an improved MR protocol, four repeated scans of sinusoidal waveforms at 6 and 10 mm displacement with 2.4, 4, and 12 s breathing periods were used.

### 2.3 Patient respiratory waveforms

Patient respiratory waveforms were selected from a database of previously acquired breathing traces recorded during clinical 4D‐CT imaging using the Varian Real‐time Position Management (RPM) system (Varian Medical Systems, Palo Alto, CA, USA). Selected waveforms were categorized into three respiratory pattern groups: regular, semiregular, and irregular.

**FIGURE 1:**
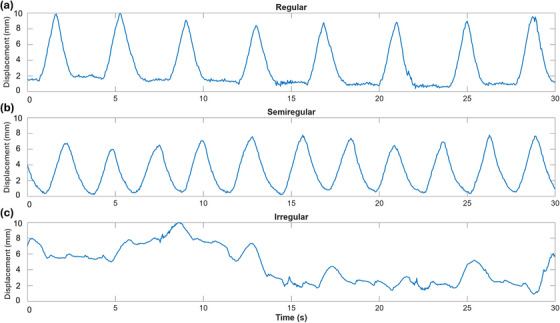
Example patient breathing waveforms for (a) regular, (b) semiregular, and (c) irregular patterns. Each waveform has been normalized and cropped to 30 s for illustration purposes.

Regular waveforms demonstrated consistent phase and amplitude throughout the acquisition, closely approximating a sinusoidal trajectory with minimal hysteresis (Figure 1a). Semiregular waveforms exhibited phase stability but demonstrated gradual amplitude drift or variation (Figure 1b). Irregular waveforms were characterized by significant deviations in both phase and amplitude, including transient breathing disruptions such as sharp inhales, coughing, and other non‐periodic behaviors (Figure 1c). Three representative waveforms of each were selected to encompass a range of breathing patterns commonly observed in the clinical setting.

Breathing waveforms were played through the QUASAR Respiratory Motion software with the specified amplitude and repeated as necessary until image acquisition was completed. Because the phantom software scales motion to the maximum amplitude of the input waveform, several irregular breathing traces exhibited transient sharp inhales that greatly exceeded the typical breathing amplitude, sometimes by more than a factor of three. Using the raw maximum amplitude from such waveforms would have compressed the majority of the respiratory cycle to low amplitudes, resulting in unrepresentative motion profiles relative to the CT. Therefore, the mean of the maximum amplitudes across breathing cycles was used for phantom programming, as it better approximates the consistent displacement measured in 4D‐CT and yields a more physiologically relevant representation of tumor motion.

Given that each waveform had a unique mean displacement amplitude, a linear mixed‐effects model was employed for statistical comparison to appropriately account for inter‐waveform variability. Imaging of the phantom was performed using dedicated 4D‐MR and 4D‐CT acquisition protocols, with sequence parameters systematically varied to evaluate their impact on displacement accuracy.

### 2.4 Image acquisition protocols

A 1.5 T MRI simulation scanner (MAGNETOM Sola, Siemens Healthineers, Erlangen, Germany) equipped with an 18‐channel phased‐array body coil was used for all MR acquisitions. Corresponding CT scans were acquired using a dedicated CT simulation system (GE Discovery RT590, GE Healthcare, Chicago, IL, USA).

A clinical 3D radial stack‐of‐stars pulse sequence (StarVIBE, Siemens Healthineers) was employed for all 4D‐MR acquisitions, with baseline imaging parameters summarized in Table 1. Slice over‐sampling was kept constant. Four acquisition parameters, slice thickness, acquisition orientation, number of radial views, and number of respiratory bins, were varied one at a time from the default sequence to assess their impact on displacement accuracy.

**TABLE 1:** MR StarVIBE clinical sequence parameters.

Parameter	Value
In‐plane resolution	1.5 × 1.5 $mm^{2}$
Base matrix size	256 ×256
Field of view (FOV)	450 ×450 mm
Number of slices	64
Slice thickness	3 mm
Slice oversampling	12.5%
Flip angle	10°
Repetition time (TR)	4.03 ms
Echo time (TE)	2.39 ms
Number of radial views	3000
Number of respiratory bins	5
Acquisition orientation	Axial, Coronal
Acquisition time	5–8 min
Fat‐water contrast	ast fat saturation

These parameters were used as a baseline for pulse sequence improvements.

StarVIBE offers amplitude‐based retrospective respiratory binning for all 4D‐MR reconstructions. Because amplitude‐based binning does not distinguish between inhalation and exhalation phases (i.e., does not explicitly account for hysteresis effects), five respiratory bins were generated for each MR acquisition, equivalent to 10 amplitude respiratory bins in CT. Ten bins were the maximum number of bins available for reconstruction on MR. CT reconstructions used phase‐based binning with ten respiratory bins, wherein post‐processing adjustments were made manually when necessary to optimize sorting quality.

This sequence has a preoptimized sequence in the axial and coronal planes, but not in the sagittal acquisition. Consequently, a sagittal acquisition was not performed in this study due to the lack of an established, vendor‐optimized sequence.

Established protocol for clinical CT scan time per imaging segment was set to the mean recorded breathing period plus an additional 1.5‐second buffer. CT images were reconstructed into a fixed ten‐phase bins using phase‐based sorting, with manual adjustments made during post‐processing to optimize bin quality. Slice thickness was set at 2.5 mm.

### 2.5 Phantom protocol evaluation

As part of the clinical protocol, patients received both an axial and coronal scan. Four key pulse sequence parameters were systematically evaluated in this study: number of radial views, number of respiratory bins, slice thickness, and the acquisition orientation. Table 3 summarizes the baseline protocol values and the tested modifications for each parameter. Baseline protocol values were determined based on what is most commonly used in clinical practice.

Each parameter was modified independently from the baseline protocol, while all other acquisition settings were held constant at the baseline value. For each modified setting, four repeated scans were acquired on both MRI and CT systems for each sinusoidal waveform amplitude.

Retrospective reconstruction into four, seven, and ten bins was subsequently performed to evaluate the influence of bin number on displacement accuracy. To further assess binning performance across different respiratory cycle characteristics, sinusoidal waveforms at 2.4‐second and 12‐second periods were also analyzed for bin number variations.

Following individual protocol modifications, the most accurate parameter value was selected based on displacement accuracy results. The coronal clinical scan was retrospectively reconstructed to account for improved accuracy. Four scans were then acquired for each waveform amplitude and period combination from both axial and coronal scans. Displacement measurements were compared between the axial 4D‐MR sequence, the coronal 4D‐MR protocol, and the clinical 4D‐CT protocol.

In addition to sinusoidal waveforms, displacement measurements were obtained for nine patient‐derived respiratory waveforms using each MR protocol and the CT protocol. For these scans, five repeated acquisitions were acquired per breathing waveform. The maximum tumor displacement, as determined using the original clinical 4D‐CT session, was used to program the mean amplitude for each corresponding patient breathing waveform. Mean amplitude values, rather than true maximum amplitudes, were selected to mitigate scaling artifacts arising from sharp amplitude spikes (i.e., coughing or deep inhales) present in some patient waveforms. Mean amplitude calculations were performed using a custom in‐house MATLAB program (MathWorks, Natick, MA, USA).

### 2.6 Phantom image analysis and statistical methods

All image datasets were exported to a commercial image analysis platform (MIM Software Inc., Cleveland, OH, USA) for displacement quantification. For each scan, the central slice in the coronal orientation was selected, and a line profile of signal intensity was drawn through the center of the sphere. Line profiles from an identical same‐coordinate line were extracted at maximum inhalation and maximum exhalation positions.

Displacement measurements were calculated using a custom in‐house Python script. The superior and inferior edges of the sphere were identified based on the full‐width‐at‐half‐maximum (FWHM) of the signal intensity curve. Absolute displacement was determined by calculating the difference between inhale and exhale profiles at both the superior and inferior edge positions.

Displacement datasets included corresponding MR and CT values for each parameter modification and waveform condition. Two reference standards were used: the known programmed ground‐truth motion amplitudes for sinusoidal waveforms and the gold‐standard clinical CT measurements. Repeated measurements were utilized to characterize run‐to‐run variability. These replicate scans were treated as independent measurements for each statistical test to improve statistical power and minimize variability.

A Welch's ANOVA followed by a Games‐Howell test was performed for each dataset.^40^ Effect sizes were estimated using η^2^ (eta‐squared) statistics. All statistical analyses and plots were generated using R software (R Foundation for Statistical Computing, Vienna, Austria). A *p*‐value of less than 0.05 was considered statistically significant. Parameters yielding no statistically significant difference or parameters closer to ground truth compared to CT measurements were considered optimal.

For analysis of scans acquired with patient‐specific respiratory waveforms, a linear mixed‐effects model (LMM) was employed to compare both MR protocol displacement measurements against the corresponding CT values, accounting for the inherent variability between different breathing patterns. Statistical significance was similarly defined at *p* < 0.05.

### 2.7 In vivo organ motion

As a part of the clinical protocol for compression‐belt patients, a 4D‐CT, axial 4D‐MR, and coronal 4D‐MR are performed. A retrospective analysis of organ motion for three compression‐belt patients with abdominal tumors was conducted to assess organ motion across modalities and identify whether an additional MR scan is necessary for improved organ displacement motion estimation.

Patients receiving a compression belt for treatment were selected due to their idealized breathing movement, which lies primarily in the superior‐inferior direction, mirroring initial phantom scans. Scans were retrospectively reconstructed into different numbers of respiratory amplitude bins. One patient's liver was outside the FOV for the axial‐MR scan, leading to the exclusion of this patient for liver displacement comparison.

Image sets were imported into MIM Software, where the maximum‐inhale and maximum‐exhale respiratory bins were rigidly registered together for each scan. Overall organ motion for the liver and kidneys was assessed. Organ motion was quantified by measuring the translational displacement of a consistent box‐based region of interest in all three anatomical directions. The resultant three‐dimensional (Euclidean) displacement was calculated for each organ. Motion trends across all three scans were visualized using R across each organ.

## 3 RESULTS

### 3.1 Phantom parameter evaluation results

#### 3.1.1 Number of radial views

**FIGURE 2:**
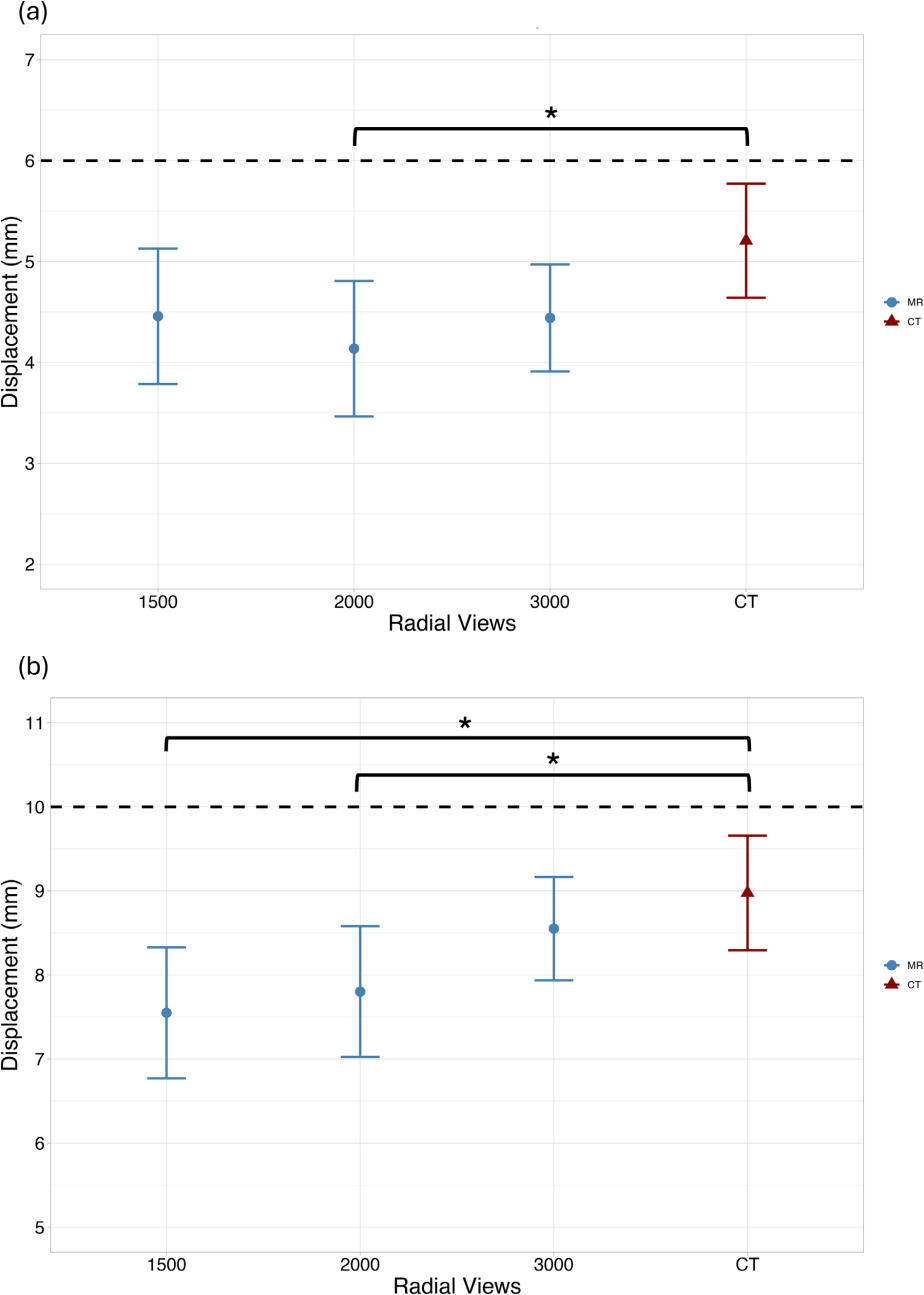
Measured displacement versus number of radial views for (a) 6 mm and (b) 10 mm ground‐truth displacement waveforms. Slice thickness = 3 mm, number of respiratory bins = 5, acquisition orientation = axial. The black dotted line denotes the ground‐truth programmed displacement (^*^ *p* < 0.05, ^**^ *p* < 0.01, ^***^ *p* < 0.001).

Results for radial view variations are summarized in Figure 2. The lowest standard error (± 0.27 mm) in displacement estimates was observed with 3000 radial views for both amplitudes. For the 10 mm ground‐truth waveform, mean displacement increased progressively as the number of radial views increased. For the 6 mm ground‐truth dataset, 3000 views had the largest displacement, but a linear trend was not seen.

Although wide confidence intervals were observed, this reflected the variability inherent in small sample sizes.

**FIGURE 3:**
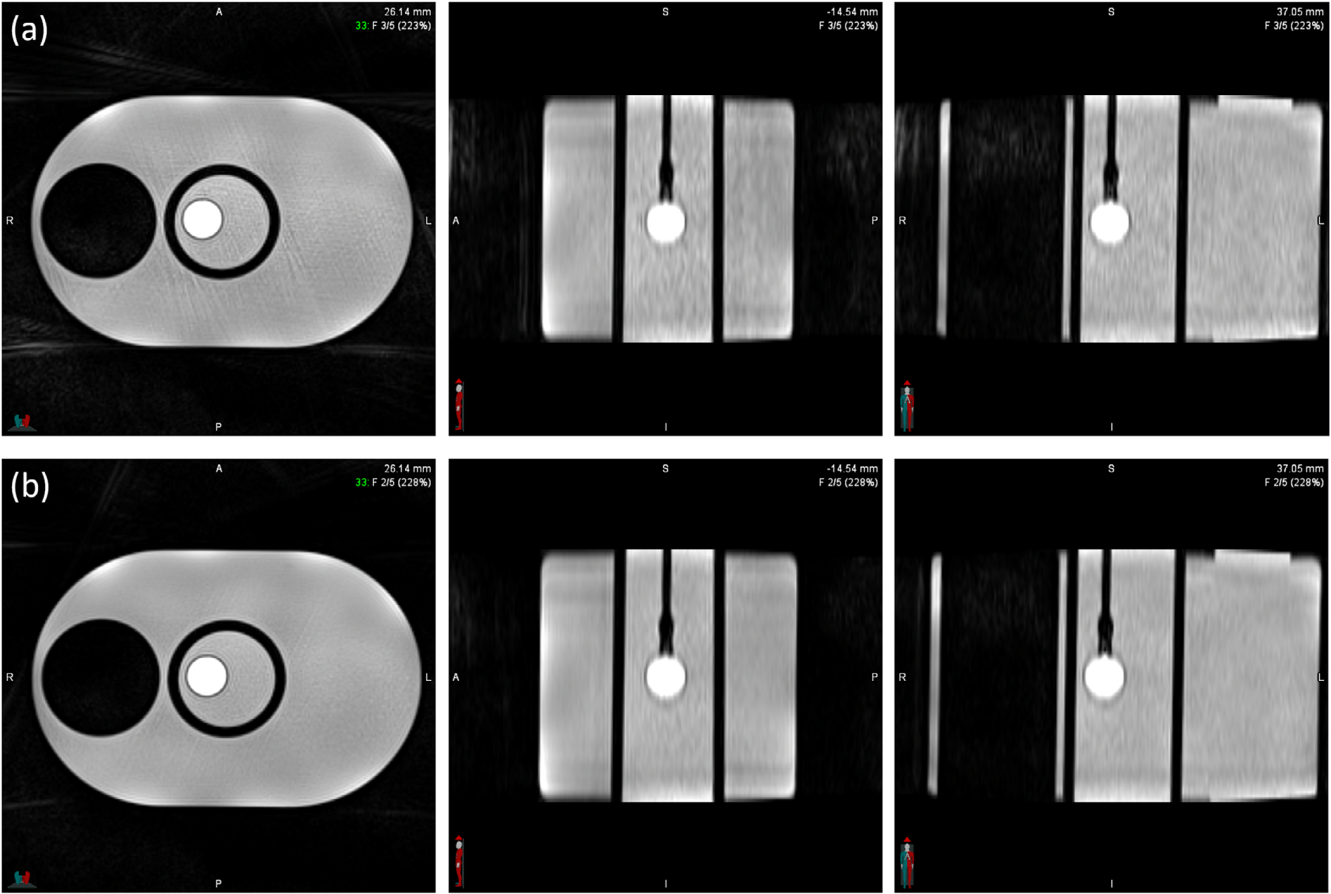
Axial, sagittal, and coronal views of an MR slice depicting streaking artifacts at (a) 1500 and (b) 3000 radial views.

Undersampling artifacts were qualitatively most prominent with 1500 radial views and decreased progressively with increasing radial views, illustrated in Figure 3. Artifacts were primarily observed in the first and last respiratory bins, which correspond to the maximum inhale and exhale positions used for displacement measurements. These streaking artifacts likely contributed to increased measurement variability and displacement error at lower sampling densities.

The 3000 radial view condition had comparable measurements to CT and thus was selected as the best parameter. It also had improved qualitative image quality with fewer undersampling artifacts. Additionally, as with other parameters, such as the number of respiratory bins, allocating views for each bin, and maximizing the overall number of views would improve other variables. Time scales linearly with the number of radial views, with scan times for 1500, 2000, and 3000 views being 3:39, 4:52, and 6:05, respectively. A longer acquisition time was considered acceptable for artifact reduction.

#### 3.1.2 Number of respiratory bins

**FIGURE 4:**
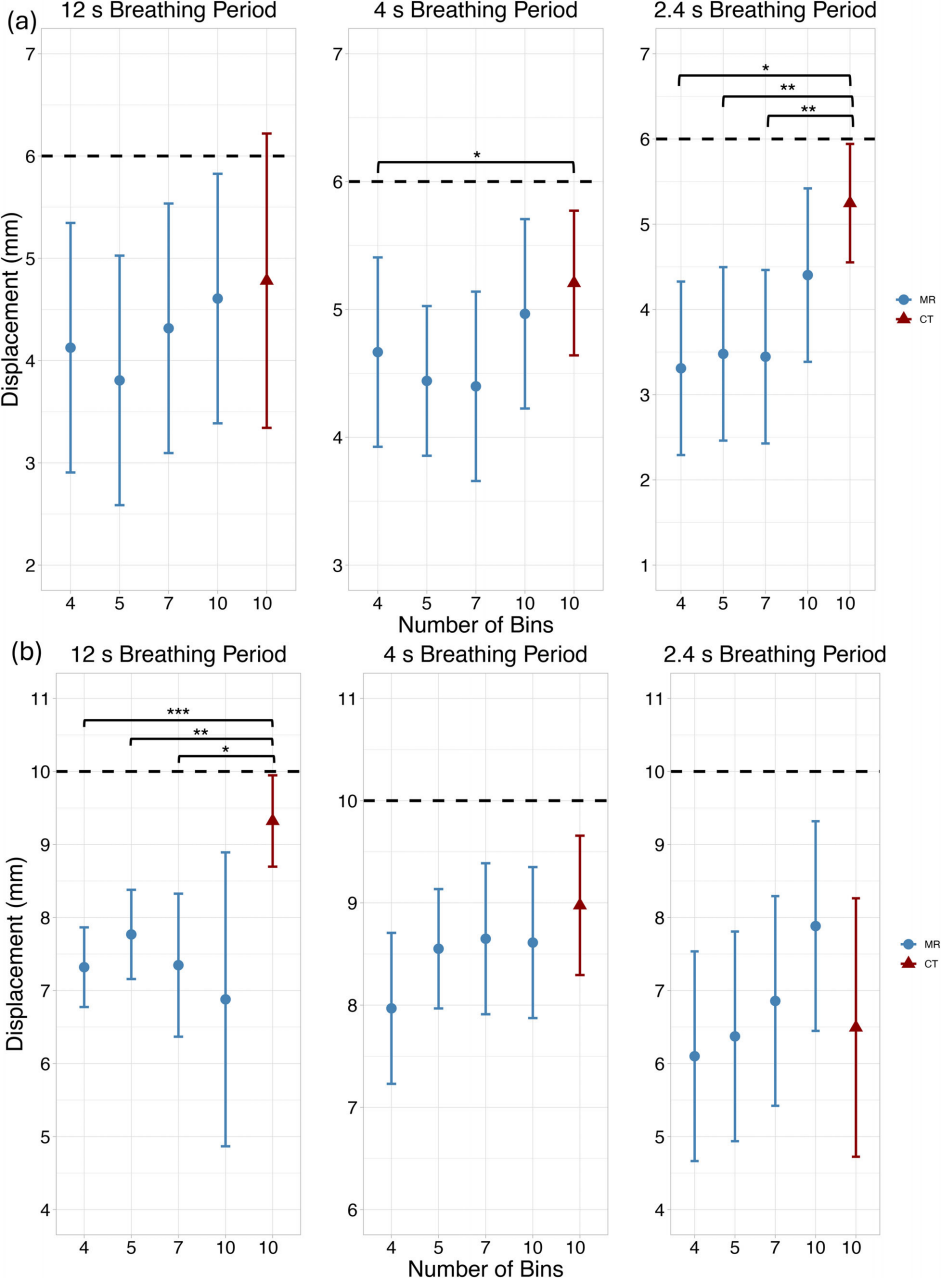
Measured displacement vs respiratory bins for (a) 6 and 10 mm (b) ground‐truth displacement across three breathing periods: 12 s, 4 s, and 2.4 s. Slice thickness = 3 mm, number of radial views = 3000, acquisition orientation = axial. The dotted line represents the programmed ground‐truth displacement. (^*^ *p* < 0.05, ^**^ *p* < 0.01, ^***^ *p* < 0.001).

Displacement measurement results for altering the number of respiratory bins are summarized in Figure 4. Both the 6 and 10 mm ground‐truth waveforms exhibited similar displacement trends across bin numbers. The most substantial discrepancies occurred for the 12‐second breathing period with 6 mm ground‐truth displacement and the 2.4‐second breathing period with 10 mm ground‐truth displacement, where three out of four bin reconstructions yielded statistically significant differences relative to CT. In contrast, for all breathing period and amplitude combinations, displacement measurements reconstructed with 10 respiratory bins most closely approximated CT‐derived measurements and consistently showed no statistically significant differences.

For the 10 mm waveform under fast breathing conditions (2.4 s period), increasing the number of respiratory bins improved MR displacement accuracy to the extent that measurements eventually outperformed CT (negative mean difference). For slow breathing periods (12 s), MR displacement measurements underestimated motion by at least 1.5 mm across all bin numbers, with no improvement observed as the number of bins increased.

Cohen's *f* values (>0.4) and η^2^ values (>0.14) were observed in most conditions, suggesting that variation in the number of respiratory bins had a substantial impact on displacement measurements. Wide confidence intervals were noted.

**FIGURE 5:**
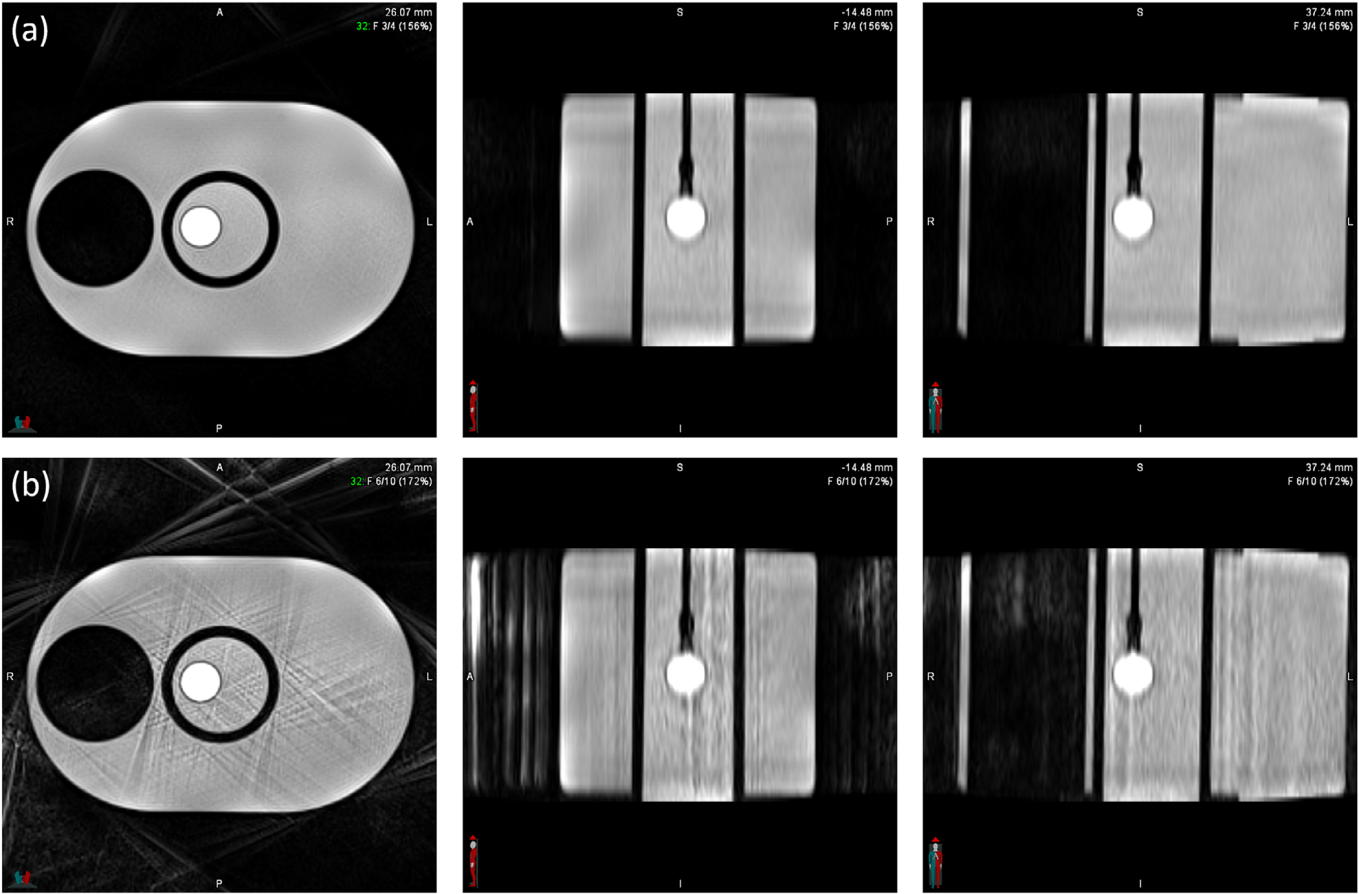
Axial, sagittal, and coronal views of images demonstrating streaking artifact severity as a function of respiratory bin number for (a) 4 bins and (b) 10 bins. Both images were acquired with 3000 radial views.

Undersampling artifacts were observed to increase with higher numbers of respiratory bins due to decreasing sampling density within the bins, particularly in the first and last bins corresponding to maximum inhalation and exhalation amplitudes (Figure 5).

Despite increased artifacts, displacement measurements were most accurate with the 10‐bin reconstruction. Based on this, 10 respiratory bins were selected for the modified 4D‐MR protocol due to measurement accuracy.

#### 3.1.3 Slice thickness

**FIGURE 6:**
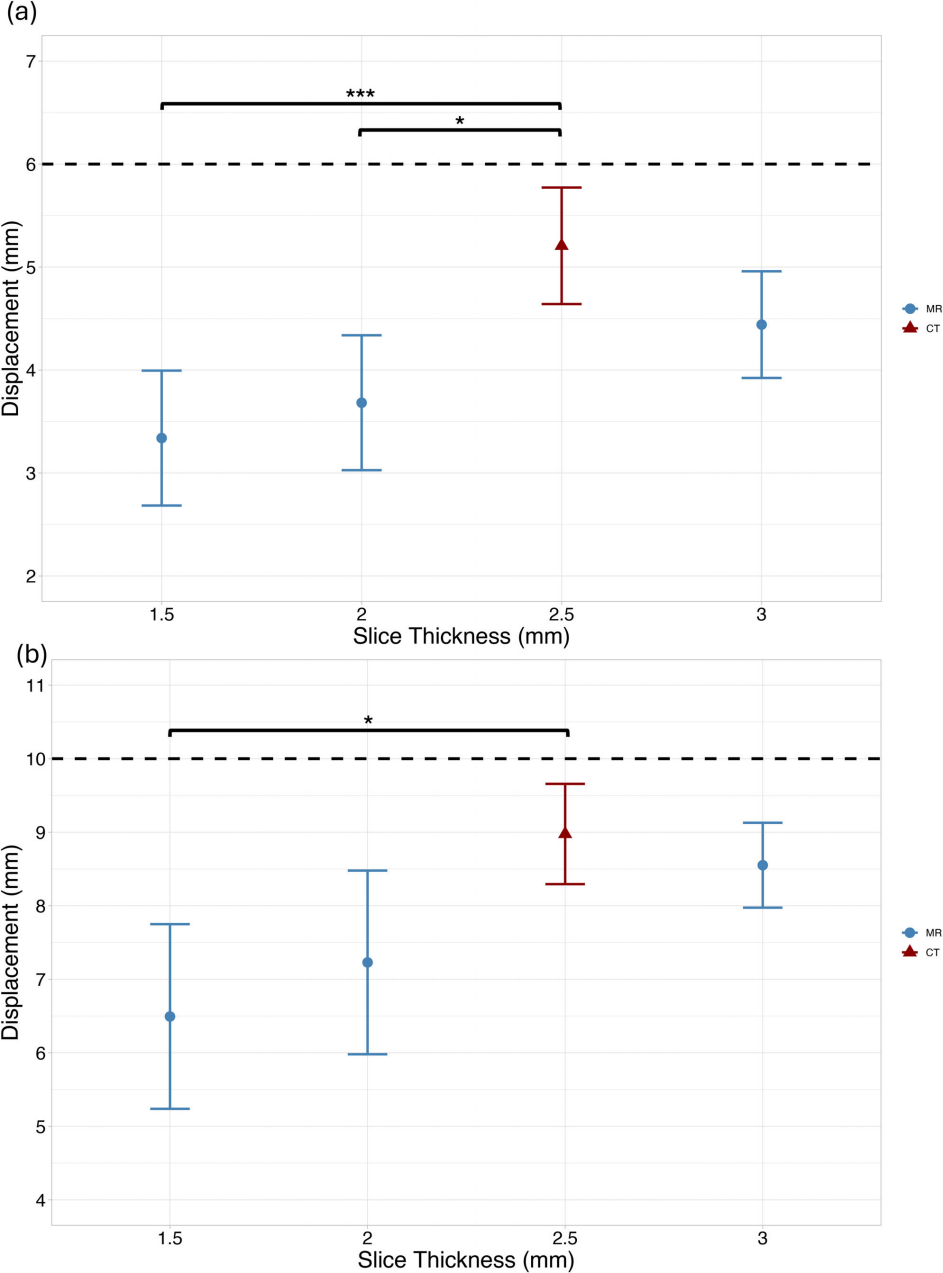
Measured displacement versus slice thickness for (a) 6 mm and (b) 10 mm ground‐truth sinusoidal waveforms. The black dotted line indicates the ground‐truth programmed displacement (^*^ *p* < 0.05, ^**^ *p* < 0.01, ^***^ *p* < 0.001).

Displacement measurements for varying slice thicknesses are shown in Figure 6. For both displacement amplitudes, measured motion amplitude increased, and standard error decreased with increasing slice thickness. The 3 mm slice thickness produced the highest mean displacement and the lowest standard error (0.77 ± 0.26 mm and 0.42 ± 0.30 mm for 6‐ and 10‐mm ground‐truth displacements, respectively) across both datasets.

Mean displacement differences relative to CT are summarized in Table 2. Statistically significant differences (*p* < 0.05) were observed for 1.5 mm slice thickness at both waveform amplitudes. A 3 mm slice thickness was comparable to CT for both amplitudes.

**TABLE 2:** MR StarVIBE pulse sequence modifications.

Parameter	Baseline Value	Tested Values
Number of radial views	3000	1500, 2000
Number of respiratory bins	5	4, 7, 10
Slice thickness	3 mm	1.5 mm, 2 mm
Acquisition orientation	Axial, coronal	Axial, coronal

From the current clinical pulse sequence, four parameters were altered. The baseline value column refers to the current clinical pulse sequence value, and the modifications column refers to the investigated values. Each parameter was changed one at a time from the current sequence for systematic evaluation.

Large Cohen's *f* values and η^2^ values were above conventional thresholds for large effects (> 0.40 and > 0.14, respectively), indicating that slice thickness had a substantial impact on displacement measurements. As in other comparisons, wide confidence intervals were observed.

**FIGURE 7:**
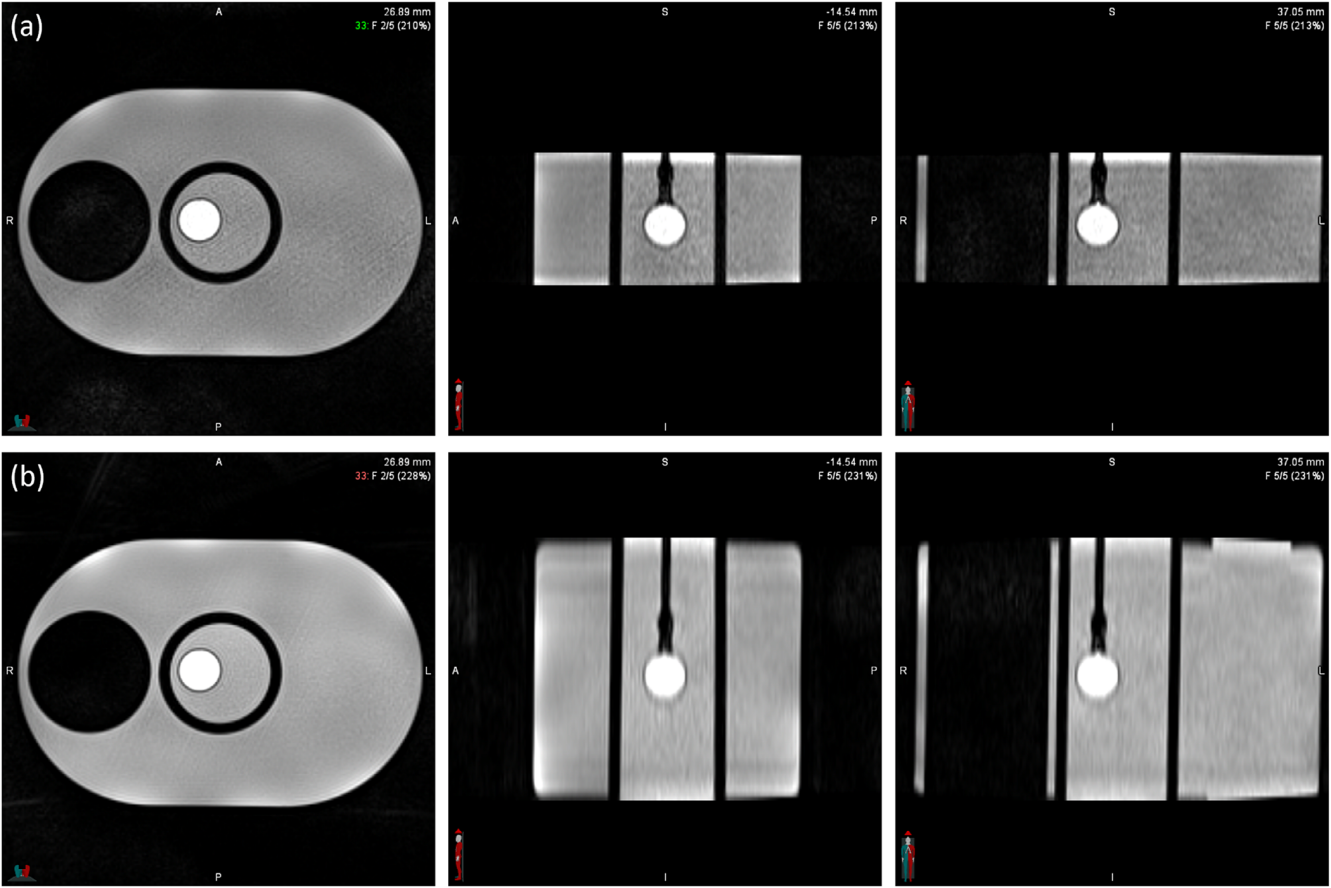
Representative Axial, sagittal, and coronal views of a slice from 4D‐MR showing phantom appearance at (a) 3 mm slice thickness and (b) 1.5 mm slice thickness. Increased sharpness and reduced motion blurring are visible in the thinner slice image.

Qualitative assessment of the coronal images revealed sharper object boundaries and reduced motion averaging with thinner slice acquisitions. 1.5 mm slices produced better edge definition, as illustrated in Figure 7. However, despite these visual improvements, 3 mm displacement measurements consistently estimated motion closer to both CT and ground truth values.

While 1.5 mm acquisitions provided qualitatively improved image sharpness, the 3 mm slice thickness was selected for the modified 4D‐MR protocol based on its superior displacement accuracy, precision, and consistency with CT‐based measurements.

#### 3.1.4 Acquisition orientation

**FIGURE 8:**
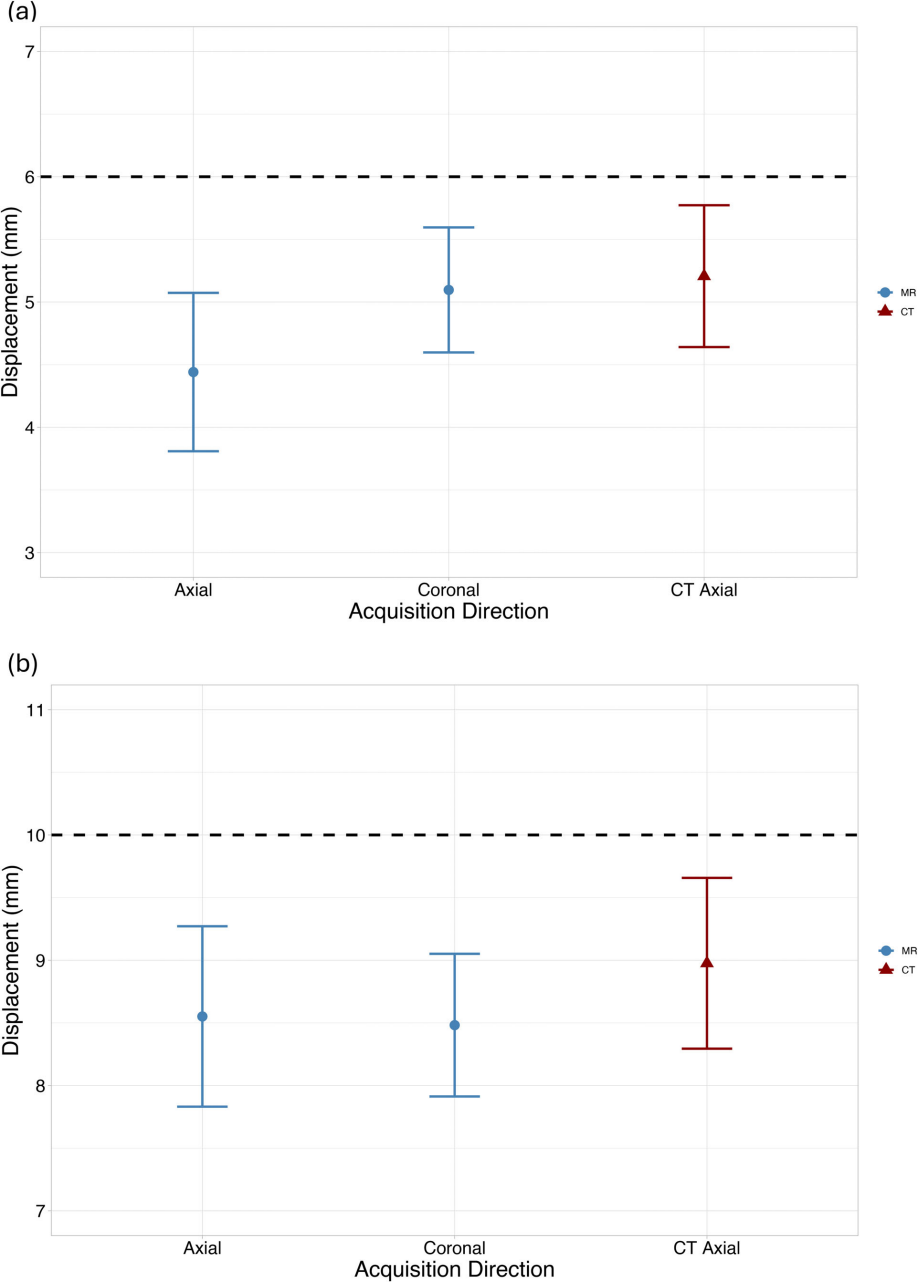
Measured displacement versus acquisition orientation for (a) 6 mm and (b) 10 mm ground‐truth sinusoidal waveforms. The black dotted line represents the ground‐truth programmed displacement. (^*^ *p* < 0.05, ^**^ *p* < 0.01, ^***^ *p* < 0.001).

Quantitative results for image acquisition orientation are presented in Figure 8. Neither acquisition direction yielded statistically different displacement results from the CT for either amplitude. However, coronal acquisitions consistently yielded lower standard deviations than axial acquisitions, suggesting improved measurement precision.

Both Cohen's *f* and η^2^ metrics indicated moderate effect sizes for acquisition orientation (∼ 0.35 and 0.10, respectively).

**FIGURE 9:**
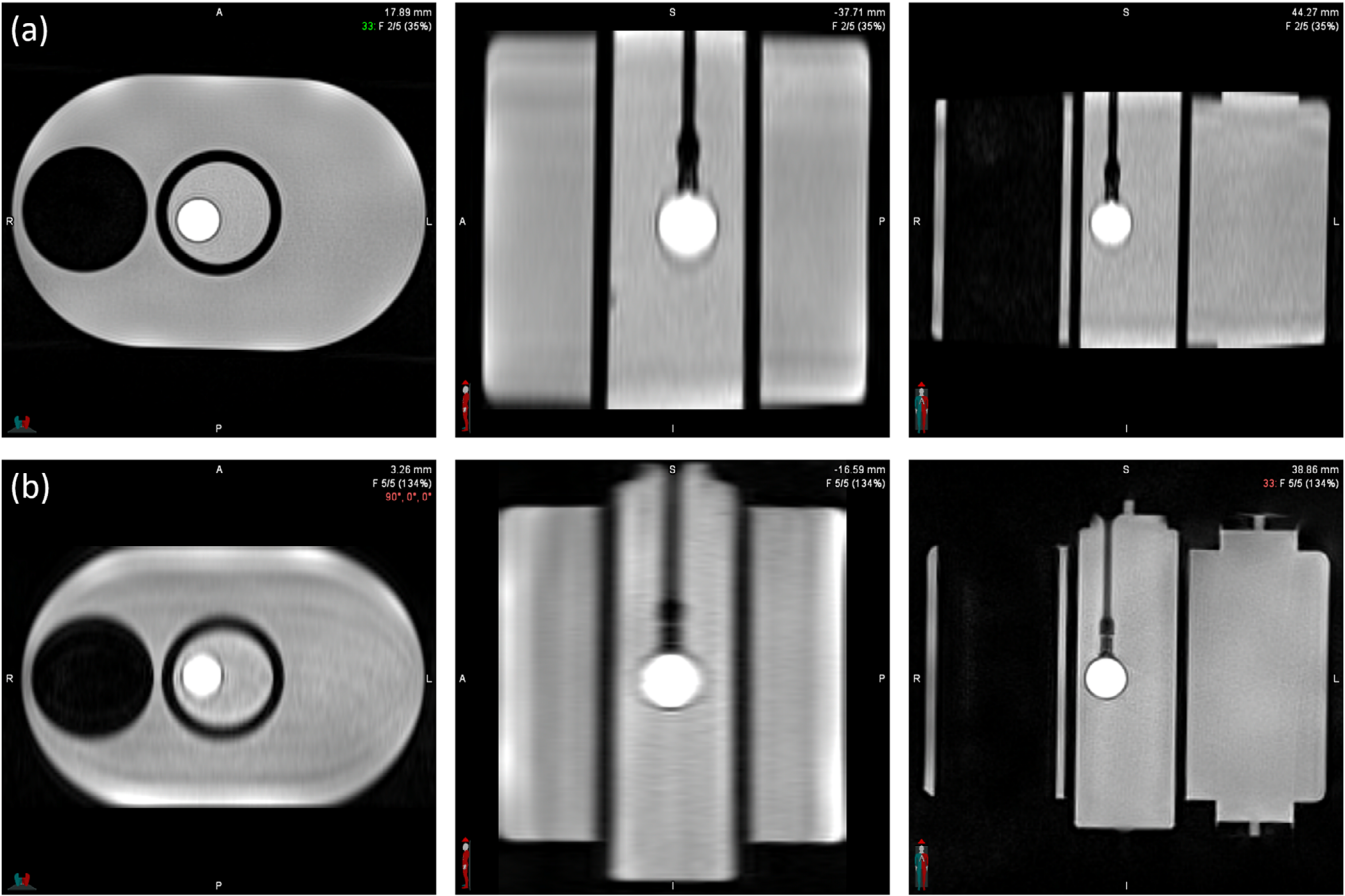
Corresponding axial, sagittal, and coronal views of an MR image for (a) axial and (b) coronal acquisition.

**FIGURE 10:**
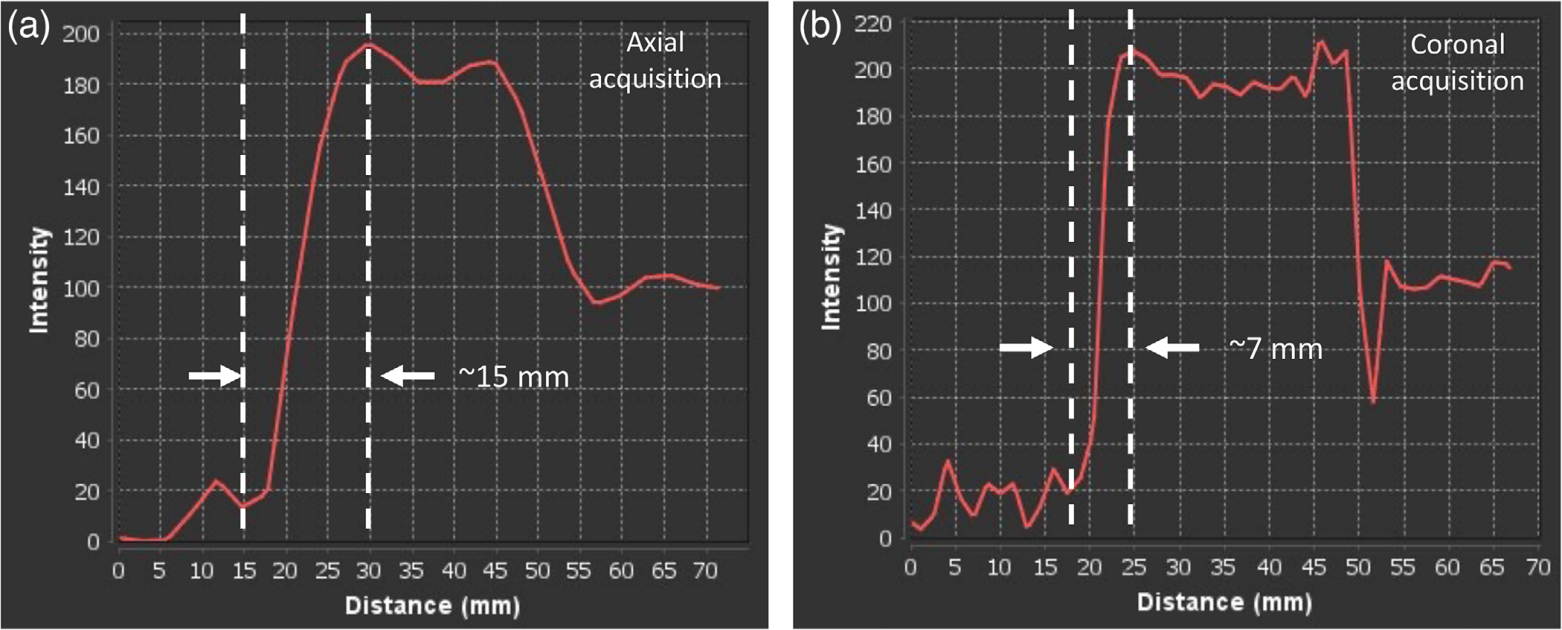
Signal intensity profiles across the moving sphere for (a) axial and (b) coronal acquisition. The coronal profile exhibits sharper edge transitions. Vertical dotted lines were included to illustrate the approximate points of measurement.

Qualitative assessment of image quality revealed sharper object boundaries and reduced motion averaging in the coronal acquisition orientation, shown in Figure 9. These improvements were further supported by signal intensity profiles. Figure 10 reveals that the coronal acquisition produced a steeper transition at object edges, resulting in a more localized signal change across the profile and improved definition of the object boundary. This narrower region for edge detection facilitated more precise displacement estimation and contributed to the reduced standard deviation observed in quantitative measurements.

Taken together, the coronal acquisition orientation demonstrated superior performance.

### 3.2 Modified 4D‐MR protocol performance on a phantom

Based on prior protocol assessment, a modified 4D‐MR protocol was constructed using 3 mm slice thickness, 3000 radial views, 10 respiratory bins, and a coronal acquisition orientation. This protocol was directly compared to the clinical axial 4D‐MR protocol and 4D‐CT across varying breathing conditions. The differing parameters between the MR protocols were acquisition orientation (axial to coronal) and the number of respiratory bins (5 bins to 10 bins).

**FIGURE 11:**
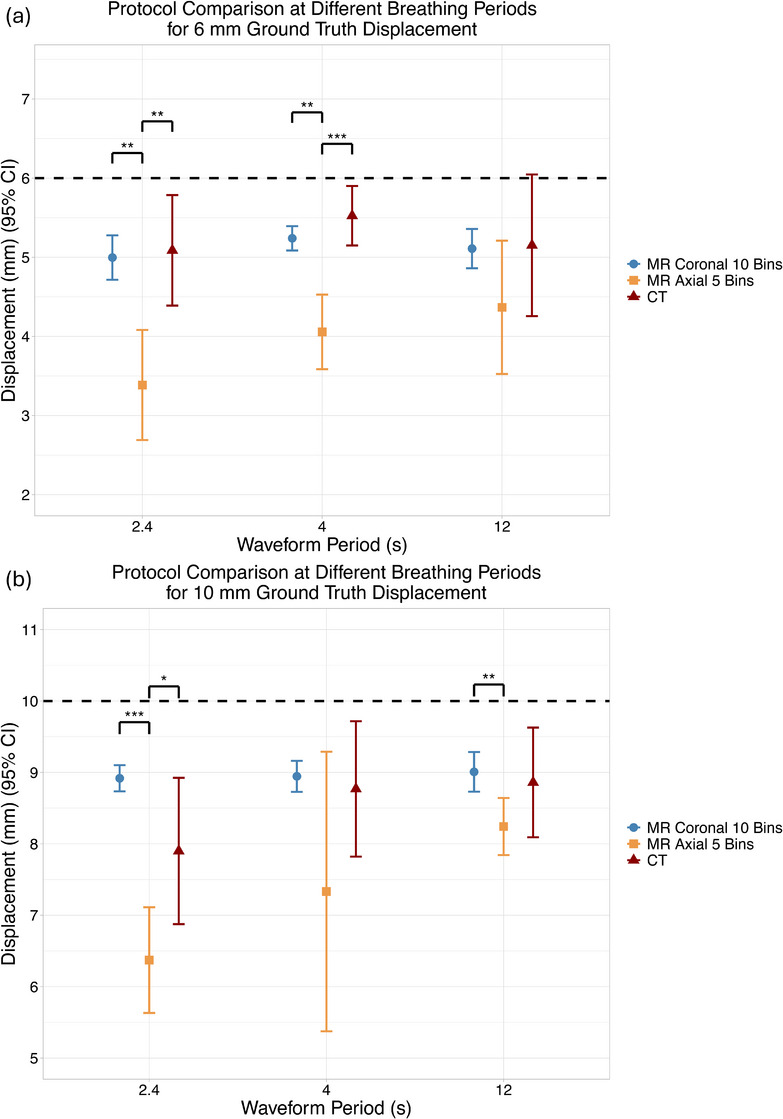
Protocol comparison at different breathing periods for (a) 6 mm and (b) 10 mm ground truth displacement. The black dotted line refers to the ground truth displacement. All values were compared with the CT clinical. (^*^ *p* < 0.05, ^**^ *p* < 0.01, ^***^ *p* < 0.001).

Displacement measurements from both MR protocols and CT were compared using sinusoidal waveforms at 6 and 10 mm ground‐truth amplitudes across three breathing periods (2.4 s, 4 s, 12 s). Figure 11 summarizes protocol performance by amplitude and period. The coronal 10‐bin MR protocol consistently produced displacement values not statistically different from CT, while the axial 5‐bin MR protocol significantly underestimated displacement in several conditions (*p* < 0.01).

For the 6 mm waveform, the coronal 10‐bin MR underestimated displacement by up to 1.70 mm, whereas the axial 5‐bin MR remained within 0.50 mm of CT in all but one condition. For the 10 mm waveform, the axial 5‐bin MR differed from CT by no more than 1 mm in any case, while the coronal 10‐bin MR underestimated displacement by more than 1 mm for the faster breathing periods.

When averaged across all breathing periods, the coronal 10‐bin MR protocol produced displacement measurements within 0.50 mm of CT for both ground‐truth amplitudes, whereas the axial 5‐bin protocol underestimated motion by more than 1.20 mm.

### 3.3 Phantom patient‐derived respiratory waveform analysis

**FIGURE 12:**
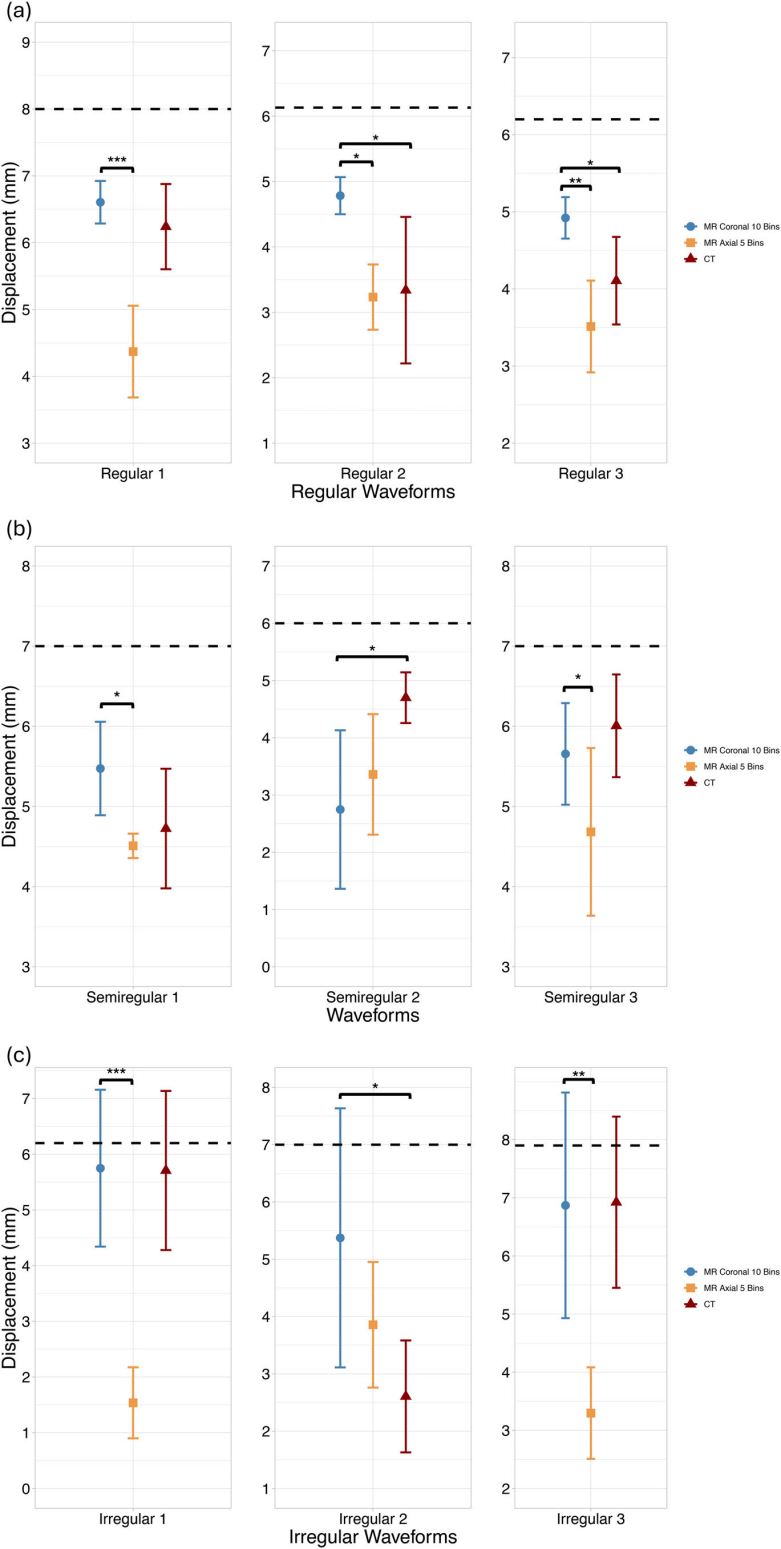
Measured displacement values by protocol for (a) regular, (b) semiregular, and (c) irregular patient‐derived respiratory waveforms. The dashed line indicates the waveform mean amplitude programmed into the phantom. Significance stars reflect comparison to MR coronal (^*^ *p* < 0.05, ^**^ *p* < 0.01, ^***^ *p* < 0.001).

Figure 12 summarizes displacement values across waveform types, and fixed‐effect estimates from the LMM with the respective standard errors and confidence intervals are outlined in Table 3.

**TABLE 3:** Linear mixed model results.

Reference condition (MR coronal = reference)	Regular (mm ± SE)	Semiregular (mm ± SE)	Irregular (mm ± SE)
MR axial	−1.73 ± 0.23^***^ (95% CI: [−2.18, −1.28])	−0.44 ± 0.31 (95% CI: [−1.05, 0.17])	−3.10 ± 0.55^***^ (95% CI: [−4.18, −2.02])
CT	−0.88 ± 0.23^***^ (95% CI: [−1.33, 0.43])	−0.52 ± 0.31 (95% CI: [−1.13, 0.09])	−0.92 ± 0.55 (95% CI: [−2.00, 0.16])
Waveform mean	1.32 ± 0.23^***^ (95% CI: [0.87, 1.77])	2.04 ± 0.31^***^ (95% CI: [1.43, 2.65])	1.01 ± 0.55 (95% CI: [−0.07, 2.09])

Displacement differences between each scan type compared to the modified MR protocol, with corresponding standard error and 95% confidence intervals

*** (*p* < 0.001).

#### 3.3.1 Regular waveforms

Displacement values for all three regular waveforms are shown in Figure 12a. The coronal 10‐bin MR protocol yielded significantly higher displacement measurements than both the axial 5‐bin MR protocol and CT for most regular waveforms (*p* < 0.001). On average, the coronal 10‐bin MR displacement was 1.73 mm greater than the axial MR and 0.88 mm greater than CT. Although the coronal MR slightly underestimated the waveform mean (by 1.32 mm), it most closely approximated true motion among the three protocols.

#### 3.3.2 Semiregular waveforms

Displacement values for each protocol are presented in Figure 12b. No statistically significant differences were observed between the coronal 10‐bin MR protocol and either the current axial 5‐bin MR or CT across most waveforms (*p* > 0.05). One exception was noted where CT outperformed both MR scans for a waveform with pronounced amplitude shift. On average, the coronal 10‐bin MR measurements were 0.44 mm greater than the axial 5‐bin MR and 0.52 mm less than CT. All protocols underestimated the waveform mean by approximately 2 mm.

#### 3.3.3 Irregular waveforms

Displacement measurements for all three protocols are shown in Figure 12c. The coronal 10‐bin MR protocol outperformed the axial 5‐bin MR protocol (mean difference: 3.10 mm; *p* < 0.001) and produced measurements most closely aligned with the waveform mean (underestimated by just 1.01 mm). The CT protocol produced slightly lower displacement values than the coronal MR (difference of 0.02 mm), but this difference was not statistically significant.

### 3.4 In vivo organ motion evaluation

Tables 4 and 5 outline organ motion displacement measurements between the maximum inhale and maximum exhale respiratory bins for compression belt patients for the liver and kidneys, respectively. Overall, the coronal 10‐bin MR scan yielded larger displacements for organ measurements in the *z*‐direction.

**TABLE 4:** In‐vivo liver displacement measurements between CT, axial 5‐bin MR (AX MR), and coronal 10‐bin MR (COR MR) 4D scans per patient.

		Liver displacements (mm)		
Direction	Modality	Patient 1	Patient 2	Mean
ΔX	AX MR	4.1	1.1	**2.6**
	COR MR	7.7	0.4	**4.1**
	CT	4.2	0.4	**2.3**
ΔY	AX MR	9.2	3.1	**6.2**
	COR MR	9.0	2.5	**5.7**
	CT	4.3	0.2	**2.2**
ΔZ	AX MR	7.0	2.7	**4.9**
	COR MR	9.7	2.4	**6.0**
	CT	16.4	0.2	**8.3**
Euclidian	AX MR	12.3	4.3	**8.3**
	COR MR	15.3	3.4	**9.4**
	CT	17.4	0.5	**9.0**

Measurements were performed in the left‐right (ΔX), anterior‐posterior (ΔY), superior‐inferior (ΔZ) directions. Euclidean distance was evaluated per patient.

**TABLE 5:** In‐vivo kidney displacement measurements between CT, axial 5‐bin MR (AX MR), and coronal 10‐bin MR (COR MR) 4D scans per patient.

		Kidney displacements (mm)			
Direction	Modality	Patient 1	Patient 2	Patient 3	Kidney mean
ΔX	AX MR	1.0	0.9	1.6	**1.1**
	COR MR	0.6	1.5	1.7	**1.2**
	CT	1.6	2.4	1.0	**1.7**
ΔY	AX MR	1.8	1.1	2.2	**1.6**
	COR MR	2.5	0.3	2.5	**1.7**
	CT	0.6	1.9	0.6	**1.0**
ΔZ	AX MR	7.6	3.1	6.3	**5.6**
	COR MR	9.6	2.4	7.0	**6.3**
	CT	5.5	1.0	4.4	**3.6**
Euclidian	AX MR	8.0	3.6	7.0	**6.1**
	COR MR	10.0	2.8	7.8	**6.8**
	CT	5.7	3.5	4.5	**4.6**

Measurements were averaged from the left and right kidney displacements. Measurements were performed in the left‐right (ΔX), anterior‐posterior (ΔY), superior‐inferior (ΔZ) directions. Euclidean distance was evaluated per patient.

Table 6 compares displacement measurements from each MR scan to the CT displacement values for both the liver and kidneys. A positive value indicates a larger CT value, and a negative value indicates a larger MR value. For both organ sets, the coronal MR had larger displacements than the CT. The axial MR showed less consistent results, though absolute differences between the axial 5‐bin MR and CT were less than 1.5 mm. All measurements were within 2.5 mm of the measured CT displacement value.

**TABLE 6:** Modality displacement differences between CT and MR.

Organ	Modality	X (mm)	Y (mm)	Z (mm)	Euclidian (mm)
Liver	CT–AX MR	−0.3	−4.0	3.4	0.7
	CT–COR MR	−1.8	−3.5	2.3	−0.4
Kidneys	CT–AX MR	0.6	−0.6	−2.0	−1.5
	CT–COR MR	0.5	−0.7	−2.7	−2.2

Two MR acquisitions were compared to CT: an axial 5‐bin MR (AX MR) and a coronal 10‐bin MR (COR MR) scan were performed on each patient. A positive value indicates that the average CT displacement measurement was larger. Conversely, a negative value indicates that the average MR measurement was larger in the specified direction. Measurements were performed in the left‐right (X), anterior‐posterior (Y), superior‐inferior (Z) directions. Euclidean distance was evaluated per patient.

**FIGURE 13:**
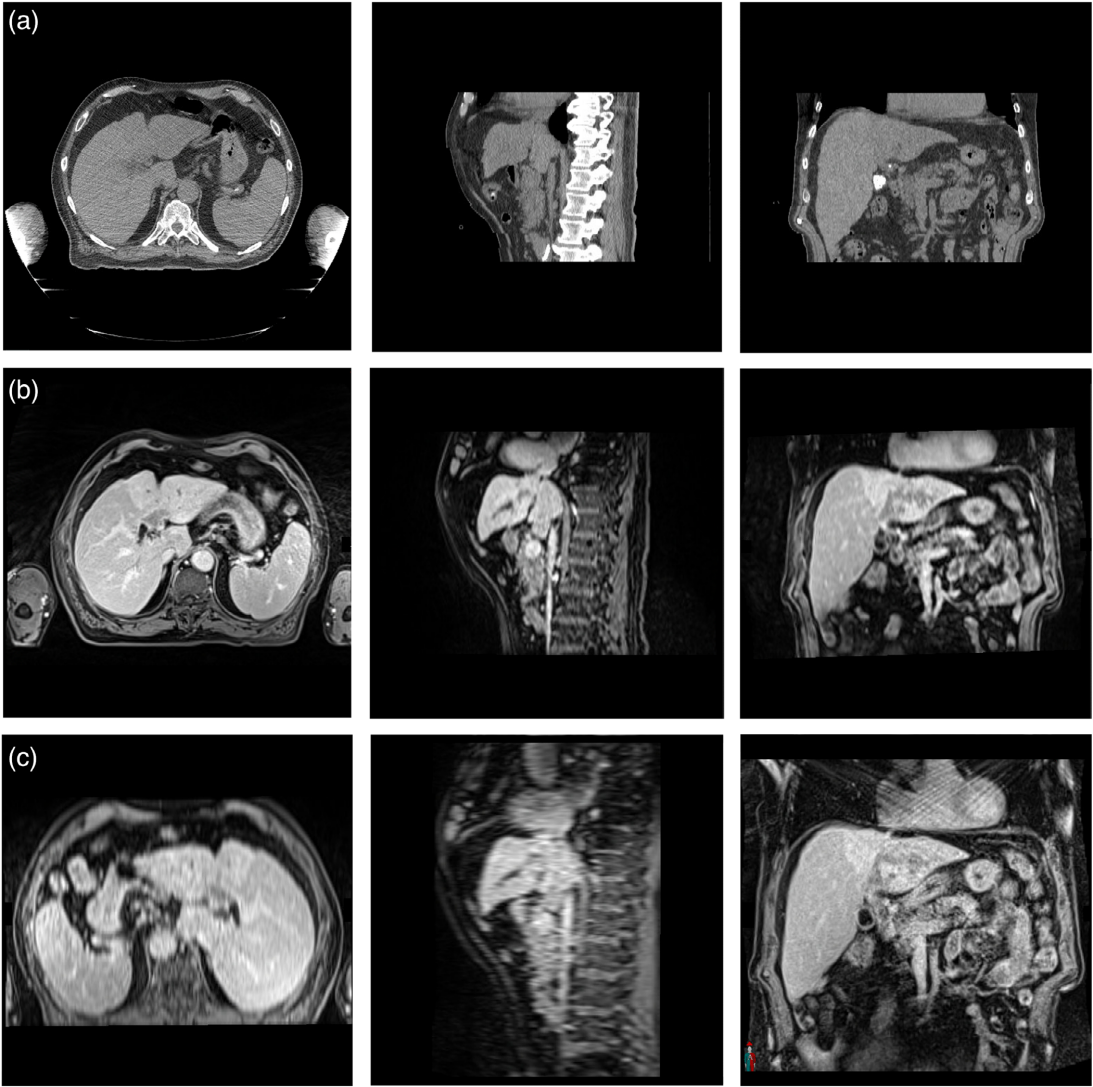
Model axial, sagittal, and coronal slices for (a) CT, (b) axial 5‐bin MR, and (c) coronal 10‐bin MR image sets for a compression‐belt patient with a liver lesion.

Figure 13 depicts the image quality differences between the CT and two MR sequences for a liver lesion. The MR scans are able to identify the liver lesion, whereas the CT contrast cannot differentiate the mass from the surrounding liver.

## 4 DISCUSSION

The results of this study demonstrate that targeted modifications to 4D‐MR acquisition parameters, specifically increased respiratory binning and coronal acquisition orientation, can significantly improve displacement accuracy relative to the clinical MR protocol. These improvements were most notable under regular and irregular respiratory conditions and brought MR measurements into close agreement with 4D‐CT across all waveform types.

Notably, in both the phantom and in vivo, the modified 4D‐MR protocol measured slightly larger motion amplitudes than 4D‐CT. This discrepancy is plausibly explained by fundamental differences in the reconstruction approaches. 4D‐CT relies on phase‐ or amplitude‐based sorting over one or two respiratory cycles, which can miss the true maximum‐inhalation and exhalation positions, particularly during irregular breathing. Additional reduced soft‐tissue contrast can further prevent accurate measurement in a clinical setting. In contrast, 4D‐MR uses amplitude‐based radial binning, where radial spokes acquired over many cycles contribute to each respiratory bin, producing a temporally averaged representation of the true extrema within the specified amplitude region. This approach reduces the likelihood of missing peak motion and, therefore, may yield slightly larger but potentially more physiologically representative displacements. Further analysis of the contribution of each parameter, as well as protocol performance across distinct waveform categories, is presented in the following sections.

### 4.1 Parameter changes with a phantom

#### 4.1.1 Number of radial views

Radial views govern the total sampling budget of the acquisition and thus set the constraint under which all other parameter decisions must operate. A low number of views amplifies the artifact burden introduced by other parameters, such as increased respiratory binning or thinner slice selection, due to fewer projections per bin or per slice. Conversely, a higher number of radial views provides more flexibility, allowing other acquisition parameters to be adjusted without significantly increasing undersampling artifacts. In this study, 3000 radial views enabled the use of ten respiratory bins while maintaining image quality and minimizing motion‐induced artifacts. Although lower view counts yielded some displacement measurements statistically comparable to CT, they were associated with greater variability and visually pronounced streaking artifacts, particularly in the bins corresponding to maximum inhalation and exhalation. This study focused on scans less than 8 min and therefore did not consider increasing the number of views beyond 3000 for clinical relevance. Scan times for 1500 and 2000 views were 3:39 and 4:52, for reference. In clinical practice, however, allowances often must be made to reduce scan time or accommodate larger fields of view, necessitating a lower number of radial views. In such cases, the impact on motion accuracy and artifact severity should be carefully balanced against clinical priorities.

#### 4.1.2 Number of respiratory bins

The number of respiratory bins directly affects the temporal resolution of motion reconstruction. In this study, displacement accuracy improved with increasing bin number, with the best agreement observed using ten bins. This finding contrasts with prior work suggesting that bin number has no statistically significant effect on displacement estimates and that a wide range of bin values may be acceptable.^35^ In our study, four out of six waveform conditions showed no significant differences between bin numbers, but for fast or irregular breathing cycles, higher bin numbers produced more accurate measurements. It is important to note that prior studies often selected lower bin numbers to minimize artifact burden, particularly radial streaking at the extremes of the respiratory cycle, which becomes more pronounced with fewer projections per bin.

The discrepancy between our findings and previous studies may be attributable to differences in the motion model, sampling strategy, or measurement endpoints. Our analysis used an idealized 1D sinusoidal motion with less than one‐third of the amplitude from previous studies to be more representative of clinical tumor displacements. Under these conditions, 10 bins provided finer amplitude resolution and more precise localization of the object in each respiratory phase. However, this benefit comes at the cost of reduced sampling per bin and increased artifacts, particularly in the end‐inhale and end‐exhale bins where motion amplitude is largest. This trade‐off may be less favorable in patient scans or in cases with more complex motion trajectories, where noise, variability, and irregularity may outweigh the benefit of finer binning. Thus, while 10 bins yielded optimal displacement accuracy in our phantom setup, the practical choice in the clinic may require balancing bin number against image quality, total scan time, and available radial views.

#### 4.1.3 Slice thickness

Slice thickness influences both the spatial resolution and the accuracy of motion depiction in 4D‐MRI. In this study, thinner slices (1.5 mm) provided visually sharper object boundaries in the coronal plane, consistent with reduced partial volume effects. However, paradoxically, displacement measurements at 1.5 and 2 mm slice thicknesses systematically underestimated the programmed ground‐truth motion compared to 3 mm slices. This discrepancy may be attributed to the sorting algorithm, but more investigation is necessary for confirmation.

These results suggest that while thinner slices improve visual detail, they may not yield more accurate displacement measurements under conditions of small amplitude 1D motion and limited SNR. For protocols aiming to quantify motion rather than visualize small structures, moderate slice thickness (e.g., 3 mm) may provide a better balance between resolution, signal fidelity, and measurement stability. Larger slice thicknesses were not investigated in this study due to radiotherapy relying on a thin slice thickness for anatomical localization information.

#### 4.1.4 Acquisition orientation

Acquisition orientation plays a critical role in displacement accuracy, particularly when motion occurs predominantly along one axis. In this study, coronal acquisitions outperformed axial acquisitions in detecting superior‐inferior (SI) motion, yielding sharper object edges and reduced variability in displacement measurements. This improvement is likely because in coronal acquisitions, SI motion occurs in‐plane, preserving edge definition and reducing motion blurring. In contrast, the same SI motion manifests as through‐plane blurring in axial acquisitions, which can obscure the object boundaries used in displacement estimation.

It is also important to note that this study focused exclusively on SI motion, as programmed in the phantom. Prior literature confirms that SI motion tends to dominate respiratory tumor displacement, particularly in the liver and pancreas. However, for tumors exhibiting substantial anterior‐posterior (AP) or left‐right (LR) motion, a coronal acquisition would be suboptimal, as those orientations would involve through‐plane motion and similar blurring artifacts to what was observed in the axial view.

While coronal acquisitions provide a technical advantage for SI motion quantification, they introduce a potential mismatch with clinical workflows. Axial imaging is the standard orientation for treatment planning, and thus axial 4D‐MR acquisitions are typically required for dose calculation and anatomical registration. Axial StarVIBE scans consistently underestimated displacement compared to both CT and coronal MR. This raises the question of whether an additional coronal 4D‐MR acquisition should be incorporated for accurate motion assessment or if axial acquisitions can be refined to produce clinically acceptable estimates.

### 4.2 MR protocol phantom comparison

#### 4.2.1 Sinusoidal waveforms

The coronal 10‐bin MR protocol displayed improved performance over the current axial 5‐bin MR protocol and equivalent or superior performance for all sinusoidal waveforms with varying periods and amplitudes. Although sinusoidal waveforms are an idealized form of a patient‐breathing waveform, this performance enhancement suggests that a coronal acquisition with more respiratory bins may more accurately track respiratory motion in a breathing surrogate.

#### 4.2.2 Phantom patient‐derived waveforms

The coronal 10‐bin MR protocol demonstrated superior performance over the axial 5‐bin MR protocol in both regular and irregular breathing conditions, with statistically significant improvements in displacement accuracy. For semiregular waveforms, all protocols showed similar performance, though displacement underestimation was more pronounced. Across all waveform types, the coronal 10‐bin MR protocol provided measurements closer to the programmed waveform mean and offered the best overall agreement with 4D‐CT.

Evaluation of patient‐derived respiratory waveforms confirmed that the coronal 10‐bin 4D‐MR protocol consistently outperformed the axial 5‐bin MR protocol in terms of displacement accuracy and precision. Displacement measurements from the axial protocol were significantly lower and more variable, particularly for regular and irregular respiratory patterns, consistent with its underperformance in phantom‐based comparisons. These results reinforce the role of acquisition orientation in motion quantification and suggest that axial MR may be insufficient as a standalone tool for motion assessment in MR‐only treatment planning workflows.

Among semiregular waveforms, results were more variable. In two of three cases, the coronal MR protocol produced displacement estimates similar to CT and axial MR, though underestimation of the programmed motion persisted. One waveform exhibited significantly lower displacement estimates from the coronal MR protocol. This waveform was characterized by increased hysteresis, reduced amplitude, and an extended exhalation phase. These features may interact unfavorably with the amplitude‐based binning strategy and artifact‐prone outer bins, reducing measurement accuracy. This result suggests that certain breathing patterns, particularly those with pronounced drift or nonuniform cycle lengths, may challenge the current implementation of 4D‐MRI and warrant further investigation.

Despite this outlier, the overall performance of the coronal MR protocol across all waveform types supports its clinical utility. Notably, 4D‐CT is known to struggle with irregular respiration, often rejecting data or producing unreliable reconstructions. In contrast, the longer acquisition time of 4D‐MR inherently averages out respiratory irregularities, leading to more stable displacement estimates. This study used looped waveforms rather than continuously acquired MR respiratory signals, exacerbating irregularities and not showing the full effectiveness of 4D‐MR's ability to average irregularities. Despite this, one of the three waveforms had a greater displacement measurement in MR than CT, showing promise of improved accuracy with irregular waveforms.

From a clinical standpoint, the observed statistical underperformance and increased variability of the axial MR protocol raise concerns for use in displacement measurements for future MR‐only treatment planning workflows. Although the axial orientation is required for dose calculation and registration, its inability to accurately resolve motion, particularly for SI‐dominant targets, may necessitate acquisition of an additional coronal 4D‐MR sequence for motion quantification. Alternatively, motion models or corrections could be applied post‐acquisition, but these would require further validation. Given that MR‐only workflows rely entirely on MR imaging for target localization and motion management, protocol optimization, including acquisition orientation, becomes essential. The results presented here support the incorporation of coronal 4D‐MR as a complementary scan to improve motion characterization, particularly for abdominal sites and dose‐sensitive populations where CT is less desirable.

### 4.3 In vivo data

Both MR scans demonstrated displacement differences within 3 mm of CT, with the coronal 10‐bin MR scan yielding measurements consistently larger in the superior‐inferior direction, agreeing with phantom data. These findings are comparable with previous patient displacement studies, though we determined this range to be up to 2 mm larger than the 0.5 mm displacement increase reported in previous studies.^35^ A contribution to this difference may arise from variations in compression belt positioning between MR and CT sessions. Small sample size, particularly with the liver measurements, prevents generalizability.

The sub‐2 mm difference observed across all organs and directions between axial 5‐bin and coronal 10‐bin MR orientations indicates that the additional coronal acquisition may provide limited additional value for motion characterization. However, there is not enough patient data to determine whether there is a significant difference between the two clinical MR scans.

An additional clinical advantage of 4D‐MR demonstrated in this study is its superior soft‐tissue contrast. In the in‐vivo case (Figure 13), the liver lesion was readily visible on 4D‐MR but indistinguishable from the surrounding tissue on 4D‐CT. This improved tumor conspicuity enables more confident target localization and may allow motion assessment based on the tumor itself rather than inferring motion from adjacent organs, as is often necessary with 4D‐CT. Although quantitative differences between tumor and organ motion were not evaluated in this study, the enhanced visualization provided by MR highlights a meaningful benefit of 4D‐MR for motion estimation beyond quantitative improvements.

### 4.4 Limitations and future directions

This pilot study was limited to 1D phantom motion using both sinusoidal and patient‐derived respiratory waveforms. While scan conditions were repeated four to five times per setting, the overall sample size was modest, particularly for the patient waveform subset. Additional repetitions and expanded waveform libraries, with more rigorous characterization of respiratory irregularities, are needed to better delineate the specific conditions under which 4D‐MRI struggles to accurately quantify motion. Additional research into hybrid amplitude and phase binning to incorporate hysteresis in this sequence may assist in cases in which motion was not adequately represented. Patient waveforms were repeated throughout the MR acquisition, which potentially reduced the effectiveness of averaging out irregular movements over a long period of time. This limitation has been noted by previous literature, citing that phantom breathing replication cannot adequately account for patient breathing irregularity.^19^, ^41^, ^42^ Additionally, the protocol was not fully optimized, as not all combinations of parameter values were investigated, nor were interplay effects studied. However, there was a significant protocol improvement.

Future work will extend these findings to healthy volunteers or patient datasets to evaluate protocol performance under more complex, multidirectional organ motion. More patients are needed to verify comparability in displacement measurements between each modality and protocol. Further investigation into artifact mitigation strategies, particularly for respiratory bins, will be essential for enabling high‐resolution motion tracking without sacrificing image quality.

Given the absence of a clinically meaningful difference between axial and coronal measurements in our preliminary studies, our institution elected to omit the coronal acquisition from the routine clinical protocol. This decision prioritized patient efficiency as the axial orientation provides superior image quality in the direction most relevant to dose calculation and target delineation. The coronal scan may be reconsidered should future work more rigorously support the implementation of comprehensive 4D‐MR workflows. Active research into the number of bins and their effectiveness is being performed retrospectively to confirm a ten‐bin protocol.

## 5 CONCLUSION

These findings support the potential clinical application of 4D‐MR as an alternative to 4D‐CT for motion assessment, though standard clinical workflows must consider the planning feasibility of these images in addition to the motion accuracy.

## AUTHOR CONTRIBUTIONS

All authors contributed to the manuscript preparation, final editing, and review. *Data acquisition*: Morgan Aire, Reagan Dugan, and Olivia Magneson. *Data interpretation*: Morgan Aire, David Solis, and Krystal Kirby. *Analysis*: Morgan Aire, Olivia Magneson, David Solis, and Krystal Kirby. *Writing and drafting work*: Morgan Aire and Krystal Kirby. *Supervision*: David Solis and Krystal Kirby. *IRB creation, intellectual content revision, and editing*: Krystal Kirby.

## CONFLICT OF INTEREST STATEMENT

The authors have no relevant conflicts of interest to disclose.

## ETHICAL REVIEW BOARD STATEMENT

This study protocol was reviewed and approved by the Institutional Review Board under IRBAM‐23‐1307 for retrospective use of imaging data and patient breathing waveforms obtained during 4D‐CT acquisition.

## Data Availability

Data can be provided upon request.
